# Assessment of strain and mechanical dyssynchrony indices in single ventricle populations by cardiac magnetic resonance feature-tracking techniques

**DOI:** 10.1186/1532-429X-15-S1-E89

**Published:** 2013-01-30

**Authors:** Ryan A Moore, Michael Taylor, Wojciech Mazur, Kan N Hor

**Affiliations:** 1 Cardiology, Cincinnati Children's Medical Center, Cincinnati, OH, USA; 2 Cardiology, The Christ Hospital, Cincinnati, OH, USA

## Background

Single ventricle (SV) physiology consists of a single pumping chamber that provides both systemic and pulmonary blood flow. The typical quantitative measurements of ventricular function (i.e. ejection fraction) cannot be accurately applied to single ventricles due to abnormal ventricular morphology. Cardiac Magnetic Resonance (CMR) has become a leading diagnostic tool in the assessment of ventricular mechanics in patients with abnormal ventricular morphology. CMR feature-tracking (FT) techniques allow for the analysis of circumferential strain (εcc), longitudinal strain (εll), and mechanical dyssynchrony indices (MDI). We hypothesize that evidence of declining εcc, εll, and MDI by CMR will be a early biomarker of ventricular dysfunction in SV patients.

## Methods

CMR data was obtained from 25 control subjects (Group A) and 30 SV patients (Group B and C). Control patients were defined as those with a clinical CMR that yielded a normal result. SV groups included patients with single right ventricles (SRV) and single left ventricles (SLV). SV patients were divided into those with normal EF ≥55% (Group B) and those with abnormal EF <55% (Group C). Standard imaging data included steady-state free precession (SSFP) short-axis cine stack images sequences. Analysis was performed using QMASS® for ventricular function and TomTec® feature-tracking for strain analysis and mechanical dyssynchrony. EF and εcc data was tabulated in the preliminary data set (εll and MDI data pending). Preliminary statistical analysis was performed via Student's t-test.

## Results

CMR data was reviewed and analyzed in 30 SV patients (mean age 17.5 ± 13.3 years) and 25 control subjects (mean age 15.6 ± 9.4 years) (p=NS). Controls (Group A) had normal EF (64.7 +/- 4.8) and εcc (-18 +/- 1.6). εcc was worse in both SV groups (B and C) compared to controls (A) (p < 0.0001). Compared to age-matched controls (A), SV patients with normal EF (Group B) had worse εcc (-14.6 +/- 2.1). SV patients with decline in EF <55% (Group C) had a continued decline in εcc (-10.6 +/- 3.7) compared to to SV patients with normal EF (p<0.01). Longitudinal strain and mechanical dyssynchrony data are currently being obtained.

## Conclusions

εcc in SV patients is abnormal despite normal EF and provides a more sensitive method of assessing subtle ventricular dysfunction in patients with abnormal ventricular morphology. Longitudinal strain and mechanical dyssynchrony indices may provide additional measures of ventricular dysfunction in SV patients based on specific type of defect and allow for further delineation of SV groups.

## Funding

No financial disclosures.

**Figure 1:**
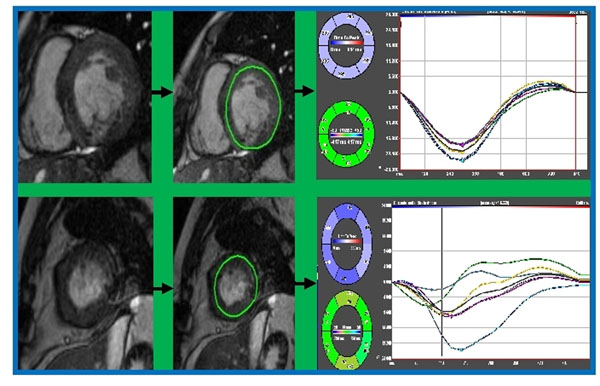
Sample images from SSFP feature tracking analysis of normal control (top panel) and SV patient (bottom panel)

